# Prediction and Analysis of the Global Suitable Habitat of the *Oryctes rhinoceros* (Linnaeus, 1758) (Coleoptera: Scarabaeidae) Based on the MaxEnt Model

**DOI:** 10.3390/insects15100774

**Published:** 2024-10-07

**Authors:** Chun Fu, Qianqian Qian, Xinqi Deng, Zhihang Zhuo, Danping Xu

**Affiliations:** 1 Key Laboratory of Sichuan Province for Bamboo Pests Control and Resource Development, Leshan Normal University, Leshan 614000, China; fuchun421@aliyun.com; 2 College of Life Science, China West Normal University, Nanchong 637002, China; qianqqqchen@foxmail.com (Q.Q.); deng.xinqi@foxmail.com (X.D.); zhuozhihang@foxmail.com (Z.Z.)

**Keywords:** *Oryctes rhinoceros*, global distribution, biological invasions, MaxEnt model, environmental factors, future scenarios

## Abstract

**Simple Summary:**

Biological invasions, such as those by the Asiatic rhinoceros beetle (*Oryctes rhinoceros*), threaten biodiversity, ecosystems, economies, and human health. This study used the MaxEnt model and ArcGIS software to predict the global distribution of this invasive beetle. The model identified areas with high suitability for the beetle mainly between 30° N and 30° S, particularly in South Asia, East Asia, Southeast Asia, and northern Oceania. Key environmental factors influencing its distribution include the amount of precipitation in the wettest month, temperatures in July and November, and precipitation in September. These findings can guide efforts to prevent and control the spread of this beetle and inform quarantine measures.

**Abstract:**

The Asiatic rhinoceros beetle, *Oryctes rhinoceros* (Linnaeus, 1758) (Coleoptera: Scarabaeidae), is a destructive invasive species that poses a serious threat to palms, oil palms, and other plants. Defining a suitable area for the distribution of *O. rhinoceros* is essential for the development of appropriate policies and preventive measures. In this work, the MaxEnt niche model and ArcGIS software were used to predict the potential geographic distribution of *O. rhinoceros* in the world based on occurrence data and related environmental variables and to speculate on the influence of environmental variables on the distribution of *O. rhinoceros*. The results showed that the suitable areas of *O. rhinoceros* beetle were mainly distributed in 30° N–30° S, and the highly suitable areas were concentrated in South Asia, East Asia, Southeast Asia, and northern Oceania. The key environmental variables that determine the distribution location of *O. rhinoceros* are Precipitation of Wettest Month (bio13), Temperature of July (tmin7), Minimum Temperature of November (tmin11), and Precipitation of September (prec9). The prediction results of the MaxEnt model can reflect the global distribution of *O. rhinoceros*. This study can provide a theoretical basis for the prevention and control of *O. rhinoceros* and the development of relevant quarantine measures.

## 1. Introduction

Predicting the potential geographic distribution of pests is one of the crucial research topics in quantitative pest risk assessment [1]. The Asiatic rhinoceros beetle, *Oryctes rhinoceros* (Coleoptera: Scarabaeidae), is a destructive invasive species that has various host plants, is parasitic on a variety of palm plants, and occasionally harms other cultivated or wild plants; among them, coconut and oil palm are the main host plants [2,3]. As important food, fiber, and wood resources, oil palm, coconut, and other palm plants have great development potential and high economic and ecological value. The occurrence of *O. rhinoceros* seriously affects the yield of crops, inhibits the growth and development of host plants, and causes the plants to wither or even die, resulting in huge losses [4].

*O. rhinoceros*is is a polyphagous pest that not only seriously harms palm plants but also feeds on sugarcane, pineapple, sisal, papaya, and other crops. When the damage is serious, it will also cause a large number of plant deaths [5]. The pest has great dispersal potential. It can reproduce in dead trunk and stump residues smoothly, spread to new settlements through the trunk tissue, and then continue to harm; the beetle can also lay eggs and survive in livestock manure accumulation sites, sawdust dump sites, and garbage dumps. These places are often neglected by people, increasing the difficulty of prevention and control [6,7]. In addition, the occurrence of natural disasters is also conducive to the reproduction of *O. rhinoceros* [8]. *O. rhinoceros* is considered a globally invasive species [9] and has invaded many countries and regions that have not previously suffered from the pest in recent years [10], including Guam (2007), Hawaii (2013), the Solomon Islands (2017), Vanuatu, and New Caledonia (2019) [11]. *O. rhinoceros* has spread all over Asia (Southeast Asia), Africa, and South Pacific Island countries, among others. The countries and regions that have experienced more serious harm include India, Mexico, Vietnam, Laos, Cambodia, Thailand, Sri Lanka, Malaysia, Singapore, Indonesia, Fiji Islands, Hawaii Islands, etc. [12,13].

*O. rhinoceros* has a long survival cycle and the phenomenon of overlapping generations. The growth and development of *O. rhinoceros* are significantly affected by climate or nutritional conditions. In general, it takes 326–455 d to complete a generation, including egg, larva, pupa, and adult. Under certain conditions, the developmental period of the egg needs 4–14 d, and the egg is white and oval as usual. The larval development period is prolonged with the increase in insect age. The developmental stages of 1st, 2nd, and 3rd instar larvae are 8–22 d, 14–28 d, and 100–156 d, respectively. The development period of the pre-pupa takes 7–18 d, and the pupa lasts for 25–41 d to reach the adult stage. The adult has a brown, smooth, and elastic dorsal surface and a brownish-red abdomen. It takes 5–29 d in the early stage of oviposition [14]. As the pest bores into the tree trunk, it is difficult for general chemicals to contact the insect’s body, so it is very difficult to control the beetle efficiently. However, studies have shown that *O. rhinoceros* can be effectively controlled by pheromones and natural enemies [3,15,16,17].

Climate changes can affect insect communities and dispersal, which in turn affect pests’ geographic distribution, numbers, and growth [1,18]. Identifying the potential distribution areas and the trend of ecological range change for invasive species is beneficial to the development of environmental management measures. Based on niche theory, the Ecological Niche Models (ENMs) analyzed the known distribution information of species and related environmental variables, constructed the model according to the corresponding algorithm to judge the ecological needs of species, and estimated and predicted the potential distribution areas of species [19,20]. Based on the principle of maximum entropy, Phillips established the maximum entropy method niche model (MaxEnt) to predict the geographical distribution of species, which can be used as a tool for statistical inference to predict the potential distribution areas of species [21]. Compared with other models, such as BIOCLIM [22], GARP [23], CLIMEX [24], and DOMAIN [25], the MaxEnt model has better prediction ability [26] and has been used to predict the potential distribution area of species in various fields [27].

Today, research on *O. rhinoceros* mainly focuses on morphological characteristics, biological habits, distribution, and harm control [14,28,29]. Prior to this, our team had already predicted the distribution of *O. rhinoceros* in China [30]. As a global invasive species [9], *O. rhinoceros* has caused great harm worldwide. Understanding the potential distribution area and change trend globally have a great effect on the prevention and control of *O. rhinoceros*. In this work, based on known distribution data and combined with environmental data, MaxEnt and ArcGIS were combined to predict the potential distribution area of *O. rhinoceros* in the world and to evaluate the potential invasion risk of *O. rhinoceros* in the future, providing the theoretical basis for the prevention and control of *O. rhinoceros* worldwide.

## 2. Materials and Methods

### 2.1. Species Distribution Data and Processing

By consulting the literature and querying the Global Biodiversity Information Facility (GBIF: https://www.gbif.org, accessed on 23 January 2024), the European and Mediterranean Plant Protection Organization (EPPO: https://www.eppo.int/, accessed on 23 January 2024), and the Commonwealth Agricultural Bureaux International (CABI: https:// www.cabi.org/, accessed on 23 January 2024), we obtained the *O. rhinoceros* distribution data. In order to reduce the influence of sampling deviation on the model accuracy, the obtained distribution point data of *O. rhinoceros* was screened using the buffer analysis method of the ArcGIS 10.8.1 software. Finally, 321 global distribution data of *O. rhinoceros* were reserved for prediction. The longitudes and latitudes of distribution points were converted into CSV format for further model analysis.

### 2.2. Bioclimatic Factors

The environmental variables used in this research were all from WorldClim (version 2.1, http://www.worldclim.org/, accessed on 26 January 2024). The environmental variables included 19 bioclimatic variables, monthly minimum temperature, monthly maximum temperature, monthly average temperature, and precipitation (Table 1), and the spatial resolution was 30 s. Environmental variables under historical climate conditions covered climate data from 1971 to 2000. The future climate data for the 2050s (2041–2060) and 2090s (2081–2100) were obtained by querying the Climate Change, Agriculture, and Food Security website (CCAFS, https://ccafs.cgiar.org/, accessed on 29 January 2024). The Intergovernmental Panel on Climate Change (IPCC) has reported four representative greenhouse gas concentration scenarios and CMIP6 has been released, so three representative concentration pathways (SSP1-2.6, SSP2-4.5, and SSP5-8.5) were selected to predict the distribution of *O. rhinoceros*.

The contribution rate of each environmental variable was detected using the jack knife test of the MaxEnt 3.4.1 software [31], and the variables with a smaller contribution were deleted [32]. The selection of climate variables has an important impact on the assessment of species habitat suitability, and there are problems, such as multi-linear repetition, among climate variables [32,33]. In this study, the extraction and analysis tool in the ArcGIS 10.8.1 software was used to execute the sampling command for the multicollinearity analysis of each climate variable, and then Pearson correlation analysis was performed on the data in R 4.4.1 software. Highly correlated variables (|r| > 0.8) were removed to eliminate the effects of multicollinearity and further improve the prediction accuracy of the models [34]. Finally, environmental variables with a high contribution rate and a low correlation coefficient were retained.

### 2.3. Parameter Settings for MaxEnt Model

The purpose of our work was to explore the influence of environmental factors on the distribution of *O. rhinoceros*. The distribution data and environmental variables of the beetle were imported into MaxEnt (version 3.4.1) to establish the initial model; 25% were randomly selected as the test set; the Jackknife method was selected to calculate the variable contribution rate; and the others were set as default values. We used the method in Section 2.2 to filter out environmental variables for model reconstruction. We selected “Make pictures of predictions” and “Do Jackknife to measure variable importance” and created a response curve. The number of repetitions was set to 10 times.

### 2.4. Suitable Area Division and Model Accuracy Evaluation

The operation results of MaxEnt 3.4.1 software were imported into ArcGIS 10.8.1 software to analyze the distribution suitability of *O. rhinoceros*. According to the partition method of assessment possibility in the IPCC report and the actual situation of *O. rhinoceros* (IPCC, 2007), habitat suitability was divided into four criteria and represented by different colors: high suitability area (0.66–1, red), medium suitability area (0.33–0.66, orange), low suitability area (0.15–0.33, yellow), and unsuitable area (0–0.15, white).

The contribution rate of bioclimatic factors was analyzed using the Jackknife test method and the Area Under the Curve (AUC) of the Receiver Operating Characteristic (ROC) curve was used to evaluate the simulation results of the model. The range of the AUC value is 0 to 1. The closer the AUC value is to 1, the higher the correlation between the prediction model and bioclimatic factors, and the higher the accuracy of the prediction results [35]. When the AUC value is less than 0.8, it indicates that the model’s performance is low. When the AUC value is in the range of 0.8 to 0.9, it indicates that the model’s performance is good. When the AUC value is in the range of 0.9–1, it indicates the excellent performance of the model.

## 3. Results

### 3.1. Verification of Model Accuracy

**Figure 1:**
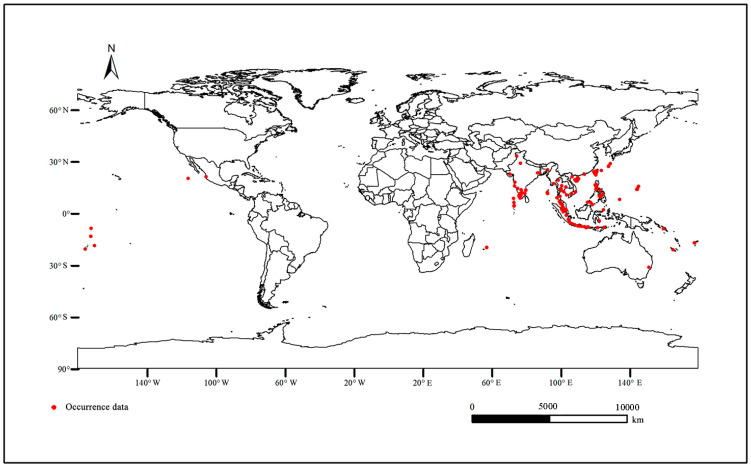
The distribution point of *O. rhinoceros* in the world. Red points, occurrence data of *O. rhinoceros*.

**Figure 2:**
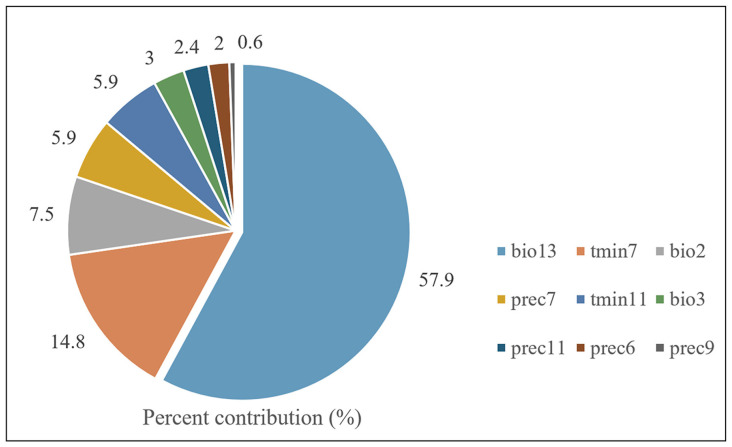
The relative contributions of the environmental variables to the MaxEnt model.

**Figure 3:**
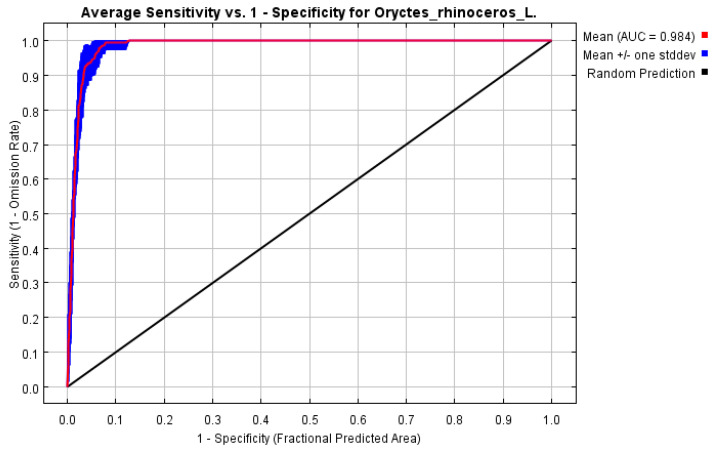
ROC curve and AUC value for the model of *O. rhinoceros*.

Climate is an important factor affecting species distribution [36]. Our study predicted the potential distribution area based on the occurrence point and environmental factors of *O. rhinoceros* (Figure 1). Combined with the Jackknife test and Pearson correlation coefficient, nine key environmental variables with high contribution rates and low correlation coefficients were screened out, including Mean Diurnal Range (bio2), Isothermality (bio3), Precipitation of Wettest Month (bio13), Precipitation of June (prec6), Precipitation of July (prec7), Precipitation of September (prec9), Precipitation of November (prec11), Minimum Temperature of July (tmin7), and Minimum Temperature of November (tmin11). Figure 2 shows the percent contribution of nine major climatic factors affecting the distribution of *O. rhinoceros*. The Pearson correlation coefficients of the above nine environmental variables are shown in Table 2. The results showed that these values were below 0.8. Based on the screened dominant climate factors, the distribution model of *O. rhinoceros* was reconstructed. As shown in Figure 3, the AUC value reached 0.984, and the accuracy of the simulation results reached an excellent standard, indicating that the constructed model can be used to predict the global suitable area of *O. rhinoceros*.

### 3.2. Current Predicted Distribution Area

**Figure 4:**
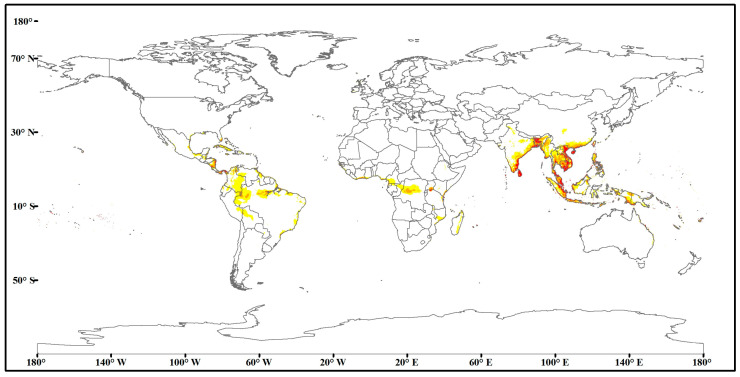
Current suitable distribution of *O. rhinoceros* in the world. Red, high suitability area with the probability of 0.66–1. Orange, medium suitability area with the probability of 0.33–0.66. Yellow, low suitability area with the probability of 0.15–0.33. White, unsuitability areas with the probability of 0–0.15.

As shown in Figure 4, according to the simulation results of nine key environmental variables and the occurrence records of *O. rhinoceros*, the current predicted distribution area of *O. rhinoceros* was obtained, and the high, medium, and low suitability grades were distinguished by different colors. The results showed that the suitable areas for *O. rhinoceros* were mainly distributed between 30° N and 30° S, including the northern part of South America, the central and southeast coasts of Africa, South Asia, East Asia, and Southeast Asia, and the northern coast of Oceania. High-suitability areas were mainly concentrated in central Africa (India, Kenya, Tanzania, and the Ivory Coast), East and Southeast Asia (Bangladesh, Indonesia, Malaysia, Thailand, and Vietnam), and Northern South America (Brazil).

**Figure 5:**
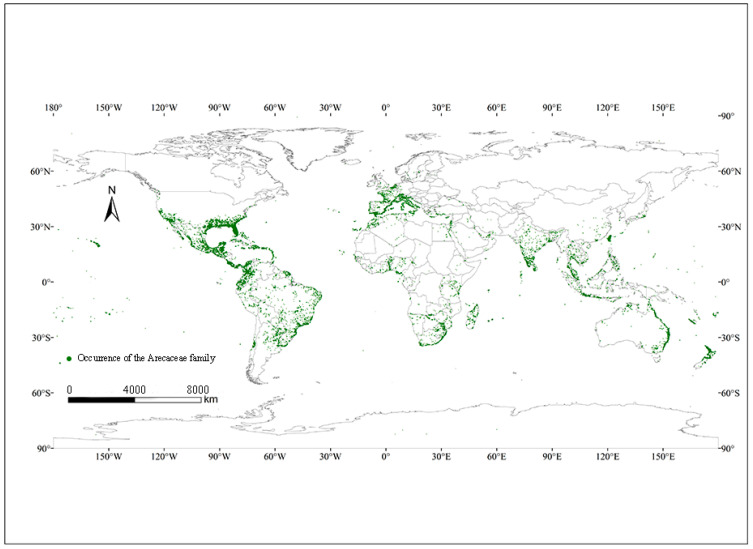
Global distribution of plants in the family Arecaceae.

As illustrated in Table 3, the total suitable area for *O. rhinoceros* in the world is currently 7.71 × 10^6^ km^2^, accounting for 5.47% of the global continental area, of which Asia is the largest, followed by South America. The high-suitability area is the largest in Asia, at 6.98 × 10^5^ km^2^, accounting for 77.63% of the total high-suitability area. The host plants of *O. rhinoceros* are plants of the Arecaceae family. Compared to the global distribution loci of plants in the Arecaceae family, the current suitable distribution areas for *O. rhinoceros* are smaller than those of the Arecaceae, but all occur within the range where plants in the Arecaceae family occur (Figure 5).

### 3.3. Potential Future Distribution of O. rhinoceros

**Figure 6:**
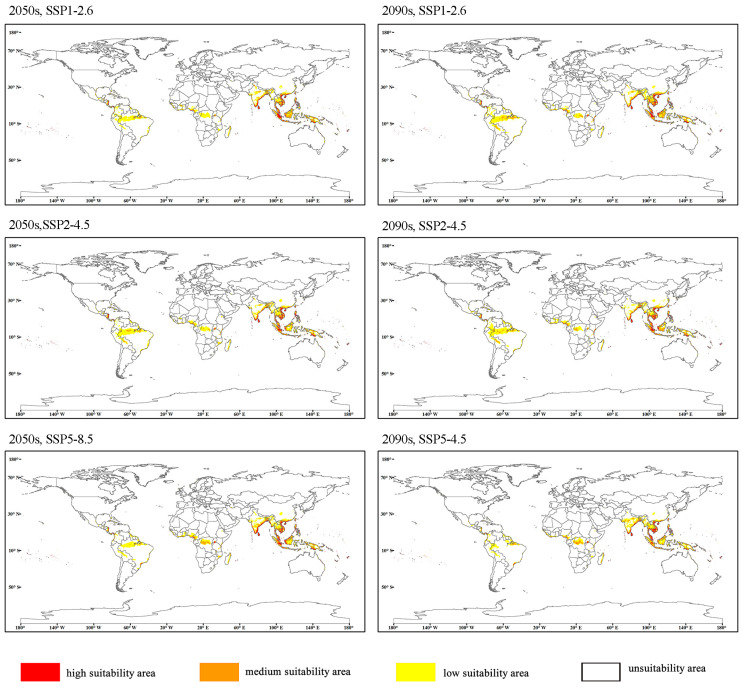
Potential distribution of *O. rhinoceros* in future period (2050s, 2090s) under the SSP1-2.6, SSP2-4.5, and SSP5-8.5 climate change scenarios.

Figure 6 showed the global suitable habitat distribution of *O. rhinoceros* predicted by the MaxEnt model under the climate scenarios of SSP1-2.6, SSP2-4.5, and SSP5-8.5 in the 2050s and 2090s, respectively. Compared with the predicted results in the current climate, the predicted areas of high, medium, and low suitability changed significantly.

High, medium, and low suitability areas showed increases in all periods except for the period 2090s under SSP5-8.5, when the area of low suitability areas decreased compared to the current period (Table 4). Under the three scenarios of the 2050s, the increased range of the high and medium suitability areas is 4.87–25.02% and 12.09–14.66%, respectively. Among them, under the high emission scenario (SSP5-8.5), the high suitability area increased by 25.02%. The trends for high and medium suitability areas were reversed for the 2050s and 2090s. The largest increase in the area of high and medium suitability areas in 2050s was in the SSP5-8.5 scenario. The largest increase in the area of high and medium suitability areas in 2050s was in the SSP1-1.6 scenario, with increases of 14.54% and 32.67%, respectively.

Compared with the current situation, there were obvious differences in the distribution of the total suitable area of each continent under different scenarios in the 2050s and 2090s. As shown in Table 5, the total suitable area in Africa expanded the most, the area in Oceania also showed an increasing trend, Europe showed an obvious shrinking trend, Asia showed an expanding trend under the SSP2-4.5 scenario in the 2090s, and South America and North America decreased under the SSP5-8.5 scenario (Table 5).

### 3.4. Environment Variables

**Figure 7:**
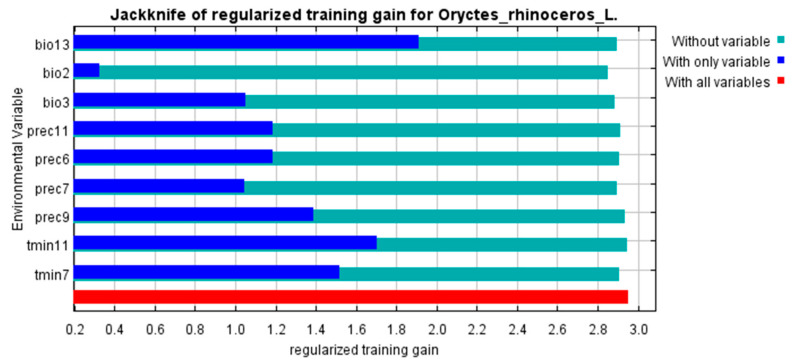
Assessing the importance of environmental variables affecting the distribution of *O. rhinoceros* using the Jackknife test.

**Figure 8:**
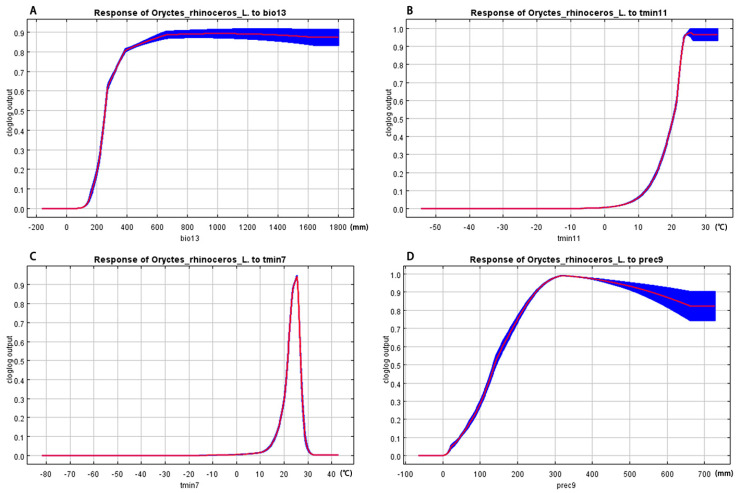
Response curve between environmental variables and probability of existence. (**A**–**D**) are bio13, tmin11, tmin7, and prec9, respectively.

The Jackknife test method was used to analyze the environmental variables that have a significant impact on the distribution of *O. rhinoceros*. The results are shown in Figure 7. All predictors affected, to a certain extent, the current distribution of *O. rhinoceros*. The nine environmental variables indicated that temperature and rainfall played an important role in predicting suitable areas for *O. rhinoceros*, of which bio13 was the most important when used alone, followed by tmin11, tmin7, and prec9. Figure 8 showed how prediction suitability changed as the value of the selected variable increased. According to the classification method of the IPCC (IPCC, 2007), the range of suitable environmental variables for the distribution of *O. rhinoceros* was divided by a threshold of 0.33. Within the appropriate range, when the value of the environmental variable was lower than the optimal value, the distribution probability was positively correlated with the environmental variable; otherwise, it was negatively correlated.

According to the response curve between environmental variables and species distribution probability in the MaxEnt model, the suitable range of environmental variables for the distribution of *O. rhinoceros* can be determined. When the value of Precipitation of Wettest Month was lower than 230.59 mm, the distribution probability of *O. rhinoceros* was lower than 0.33, the distribution probability gradually increased with the increase in precipitation, and the optimal value was 667.99 mm. When the value of Minimum Temperature of November was higher than 17.91 °C, it was suitable for the distribution of *O. rhinoceros*, and the most suitable temperature was 25.51 °C. When the value of the Minimum Temperature of July was in the range of 20.39–25.40 °C, with the increase in temperature, the predicted distribution probability of *O. rhinoceros* increased rapidly, and decreased rapidly when the temperature was higher than 25.40 °C; the suitable range was 20.39–27.47 °C. The value of the Precipitation of September was lower than 109.38 mm, and the distribution probability was lower than 0.33. When the value of the Precipitation of September increased to 322.76 mm, it reached the maximum suitable probability of 0.99.

## 4. Discussion

ENMs are important ecological research tools that are usually used to predict the impact of climate change on species distribution [37,38]. In the past 20 years, ENMs have developed rapidly. Currently, there are more than 33 niche models, and new algorithms are constantly being introduced in those models [39]. The MaxEnt model has the advantages of stable operation results and relatively convenient use [40]. It has low requirements in sample size and can fit complex models from small data sets, and the accuracy of the model will improve with the increase in sample size [21,41]. Elith et al. illustrated that when the prediction abilities of various niche models were compared, the Maxent model usually showed the best prediction performance [27]. Furthermore, numerous studies have declared that when comparing the prediction accuracy of commonly used niche models, the MaxEnt model has a better prediction effect [42,43,44]. At present, the MaxEnt model is widely used around the world [45]. Consequently, MaxEnt was typically chosen as one of the best simulation programs for species prediction. This study was based on the MaxEnt model to simulate the current and future potential global geographic distribution of *O. rhinoceros*. The performance and reliability of the MaxEnt model were evaluated by using the AUC method, which is currently a universal metric for evaluating diagnostic test results [46], and the results showed that the model could be used to simulate the potential distribution of *O. rhinoceros*.

Research has shown the impact of climate change on pest expansion [47]. As an invasive species, *O. rhinoceros* has now expanded to Oceania, the Americas, and the African continent [48]. The study predicted that the spread of *O. rhinoceros* will be more serious, which will undoubtedly cause greater losses to the ecological environment and forestry economy. Research on the potential distribution and range of pests caused by climate change is the key to scientific management and control decisions. The prediction results of the MaxEnt model declared that all continents except Antarctica were suitable for the growth of *O. rhinoceros*, mainly concentrated in tropical and subtropical countries where the climate was characterized by abundant precipitation, high temperature, and humidity. The high-suitability areas were mainly concentrated in South Asia, East Asia, Southeast Asia, and northern Oceania, and the high and middle suitability areas will show an obvious expansion trend in the future. This was consistent with previous related studies on this species [49]. However, when comparing the results of future changes in the suitable area, we found in our conclusion that the suitable area will expand in the future, but the results of Hao et al. showed a contraction. This may be related to the different contextual models we used. The SSP used in this paper is the latest released scenario, which is more informative. If certain areas are predicted to be highly suitable areas for *O. rhinoceros* and the beetles do occur, the agricultural departments should strengthen control work in these areas; if the region has not yet been simulated as a highly suitable area for the Asiatic rhinoceros beetle’s survival, the agricultural departments should focus on strengthening quarantine work to prevent its invasion.

The occurrence and spread of pests are strongly related to environmental factors, host factors, and agroecosystems. Other things being equal, climatic factors may be the key factor influencing the widespread distribution of pests [50,51]. In this study, the importance of the variables was determined using the Jackknife test method, and the most important environmental variables affecting *O. rhinoceros* mainly included bio13, tmin11, tmin7, and prec9. The response curves indicated that the probability of the presence of *O. rhinoceros* was extremely low when the Minimum Temperature of November was below 17.91 °C, indicating that low temperature was the key factor affecting the distribution of *O. rhinoceros* (Figure 8B). The range of suitable values for the Minimum Temperature of July was small (20.39–25.40 °C), which indicated that *O. rhinoceros* was highly sensitive to extreme temperature changes (Figure 8C). Bedford’s (1980) study showed that temperature affected the growth, reproduction, and distribution of *O. rhinoceros*, with a suitable range of 27–29 °C for larval development. Ainslie’s (1930) investigation found that *O. rhinoceros* was more sensitive to low temperatures than some insects. This is consistent with the results of our work (Figure 8B,C). Furthermore, lots of studies have shown that environmental humidity can affect the growth and development of insects [52,53,54]. The tolerance to humidity was relatively high, and *O. rhinoceros* can develop normally at a relative humidity of 85–95% [12], and the distribution of *O. rhinoceros* was positively correlated with humidity [55]. In addition, humidity was one of the important factors in the search for breeding sites for *O. rhinoceros* [56], and rainfall affected the foraging activity of adults at night [57]. The conclusion of this study pointed out that when the Precipitation of Wettest Month and Precipitation of September were higher than 230.59 mm and 109.38 mm, respectively, it was suitable for the distribution of *O. rhinoceros* (Figure 8A,D), which indicated that *O. rhinoceros* preferred to be distributed in environments with higher humidity within a certain range. This is consistent with the findings of some earlier studies. Overall, precipitation and temperature together limit the distribution pattern of *O. rhinoceros*.

The MaxEnt model has advantages in predicting the potential distribution of species, but there are still some limitations [34]. This study used limited occurrence data, only selected the corresponding environmental variables, and used the MaxEnt model to predict the global habitat of *O. rhinoceros*. The distribution of the species was not only limited by environmental conditions but also affected by other factors, such as interspecific relationships, human activities, altitude, and host plants [58]. Therefore, biotic and abiotic factors, such as human activities, hosts, etc., can be incorporated into the model when studying the suitable habitat of *O. rhinoceros* in the future, and further studies can be carried out in combination with more ENMs.

## 5. Conclusions

In this work, the MaxEnt model and ArcGIS technology were combined to simulate the global suitability area of the Asiatic rhinoceros beetle, *Oryctes rhinoceros*, by using distribution point information and climate factors. The results showed that the most important environmental variables driving the global distribution of *O. rhinoceros* were Precipitation of Wettest Month (bio13), Minimum Temperature of July (tmin7), Minimum Temperature of November (tmin11), and Precipitation of September (prec9). The suitable area was mainly distributed in 30° N–30° S, and the distribution range of *O. rhinoceros* in the middle and the high suitability areas showed a trend of further expansion. The relevant departments should take appropriate control measures to strengthen the sustainable management of invasive species. Our study will expand the understanding of the environmental drivers of the distribution of *O. rhinoceros* and provide a reference for the prevention and control of potential invasive areas of *O. rhinoceros* transmission or potential invasion.

## Figures and Tables

**Table 1:** Climatic variables used for predicting potential geographic distribution of *O. rhinoceros*.

Code	Environmental Variables
bio1	Annual Mean Temperature
bio2	Mean Diurnal Range (Mean of monthly (max temp–min temp))
bio3	Isothermality (bio 2/bio 7) (×100)
bio4	Temperature Seasonality (standard deviation × 100)
bio5	Max Temperature of Warmest Month
bio6	Min Temperature of Coldest Month
bio7	Temperature Annual Range (bio5-bio6)
bio8	Mean Temperature of Wettest Quarter
bio9	Mean Temperature of Driest Quarter
bio10	Mean Temperature of Warmest Quarter
bio11	Mean Temperature of Coldest Quarter
bio12	Annual Precipitation
bio13	Precipitation of Wettest Month
bio14	Precipitation of Driest Month
bio15	Precipitation Seasonality (Coefficient of Variation) 1
bio16	Precipitation of Wettest Quarter
bio17	Precipitation of Driest Quarter
bio18	Precipitation of Warmest Quarter
bio19	Precipitation of Coldest Quarter
Tmin	Minimum Temperature of Each Month
Tmax	Maximum Temperature of Each Month
Tmean	Mean Temperature of Each Month
Prec	Precipitation of Each Month

**Table 2:** Pearson correlation coefficients of crucial environmental factors.

	bio2	bio3	bio13	prec6	prec7	prec9	prec11	tmin7
bio3	0.140							
bio13	−0.181 *	0.039						
prec6	−0.233 **	−0.227 **	0.782 **					
prec7	−0.110	−0.279 **	0.782 **	0.776 **				
prec9	−0.210 **	−0.126	0.480 **	0.640 **	0.700 **			
prec11	−0.184 *	0.638 **	0.030	−0.279 **	−0.374 **	−0.130		
tmin7	−0.324 **	−0.231 **	0.068	0.160 *	0.173 *	0.343 **	0.002	
tmin11	−0.418 **	0.557 **	0.084	−0.008	−0.079	0.093	0.490 **	0.457 **

** Significantly correlated at *p* < 0.01 level. * Significantly correlated at *p* < 0.05 level.

**Table 3:** Projections of the current suitable areas of *O. rhinoceros* (×10^4^ km^2^).

Continent	Low Suitability	Medium Suitability	High Suitability	Total Suitability Area
Africa	122.57	46.75	14.41	183.73
Asia	118.38	118.51	69.79	306.68
Europe	2.37	0.10	0.04	2.51
North America	29.66	16.27	2.70	48.63
Oceania	12.63	8.06	1.98	22.67
South America	179.63	26.78	0.98	207.39
Total	465.24	216.47	89.90	771.61

**Table 4:** The difference of suitable areas between current and future climatic conditions.

Decade	Scenarios	Predicted Suitable Areas (×10^4^ km^2^)			Comparison with Current (%)		
		Low Suitability	Medium Suitability	High Suitability	Low Suitability	Medium Suitability	High Suitability
Current		465.24	216.47	89.90			
2050s	SSP1-2.6	494.59	243.70	94.28	6.31%	12.58%	4.87%
	SSP2-4.5	518.62	242.64	95.49	11.47%	12.09%	6.22%
	SSP5-8.5	486.06	248.21	112.39	4.48%	14.66%	25.02%
2090s	SSP1-2.6	508.73	287.20	102.97	8.55%	32.67%	14.54%
	SSP2-4.5	519.56	261.56	95.02	11.68%	20.83%	5.70%
	SSP5-8.5	431.53	244.96	94.68	−7.25%	13.16%	5.32%

**Table 5:** The current and future changes of total suitable area under different scenarios.

Continent	Suitable Area (×10^4^ km^2^)						
	Current	2050s			2090s		
		SSP1-2.6	SSP2-4.5	SSP5-8.5	SSP1-2.6	SSP2-4.5	SSP5-8.5
Africa	183.73	235.00	218.59	262.49	221.23	207.78	250.10
Asia	306.68	327.49	335.19	342.04	359.25	333.12	328.15
Europe	2.51	1.85	1.56	3.60	0.78	3.28	1.87
North America	48.63	32.75	32.22	25.43	28.32	34.20	20.06
Oceania	22.67	34.04	30.44	32.74	31.89	31.32	31.45
South America	207.39	193.98	231.29	173.15	249.97	259.00	132.22
Total	771.61	825.11	849.29	839.45	891.44	868.70	763.85

## Data Availability

The data supporting the results are available in a public repository at: https://doi.org/10.5281/zenodo.5774066 (accessed on 12 January 2024).
